# 4,4-Difluoro-1,3,5,7-tetra­methyl-8-penta­fluoro­phenyl-4-bora-3a,4a-diaza-*s*-indacene

**DOI:** 10.1107/S1600536810003703

**Published:** 2010-03-06

**Authors:** XiaoFeng Zhou

**Affiliations:** a College of Transportation, Southeast University, Nanjing 210096, People’s Republic of China

## Abstract

In the title dye compound, C_19_H_14_BF_7_N_2_, the boron–dipyrromethene core lies on a crystallographic mirror plane which bis­ects the BF_2_ and penta­fluoro­phenyl groups. The dihedral angle between the penta­fluoro­phenyl ring and the tricyclic system is thus 90° by symmetry. The *sp* ^3^-hybridized B atom has a slightly distorted tetra­hedral coordination.

## Related literature

For boron–dipyrromethene (BODIPY) dyes, see: Bergström *et al.* (2002 ▶); Trieflinger *et al.* (2005 ▶). For geometrical parameters in other BODIPY-based compounds, see: Picou *et al.*(1990 ▶); Wang *et al.*(2007 ▶); Kuhn *et al.* (1990 ▶).
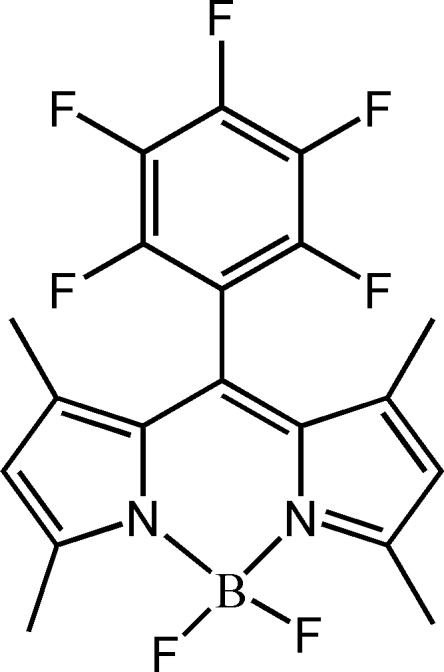


## Experimental

### 

#### Crystal data

C_19_H_14_BF_7_N_2_
*M* *_r_* = 414.13
Monoclinic, 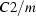

*a* = 12.4060 (5) Å
*b* = 7.5490 (9) Å
*c* = 19.720 (3) Å
β = 97.12 (2)°
*V* = 1832.6 (4) Å^3^
*Z* = 4
Mo *K*α radiation
μ = 0.14 mm^−1^
*T* = 293 K
0.2 × 0.2 × 0.2 mm

#### Data collection

Rigaku SCXmini diffractometer
Absorption correction: multi-scan (*CrystalClear*; Rigaku, 2005 ▶) *T*_min_ = 0.973, *T*_max_ = 0.979
8232 measured reflections
1936 independent reflections
1585 reflections with *I* > 2σ(*I*)
*R*_int_ = 0.032

#### Refinement

*R*[*F* ^2^ > 2σ(*F* ^2^)] = 0.056
*wR*(*F* ^2^) = 0.162
*S* = 1.06
1936 reflections
170 parameters
H-atom parameters constrained
Δρ_max_ = 0.24 e Å^−3^
Δρ_min_ = −0.23 e Å^−3^

### 

Data collection: *CrystalClear* (Rigaku, 2005 ▶); cell refinement: *CrystalClear*; data reduction: *CrystalClear*; program(s) used to solve structure: *SHELXS97* (Sheldrick, 2008 ▶); program(s) used to refine structure: *SHELXL97* (Sheldrick, 2008 ▶); molecular graphics: *SHELXTL* (Sheldrick, 2008 ▶); software used to prepare material for publication: *SHELXL97*.

## Supplementary Material

Crystal structure: contains datablocks I, global. DOI: 10.1107/S1600536810003703/bh2269sup1.cif

Structure factors: contains datablocks I. DOI: 10.1107/S1600536810003703/bh2269Isup2.hkl

Additional supplementary materials:  crystallographic information; 3D view; checkCIF report

## Figures and Tables

**Fig. 1.**
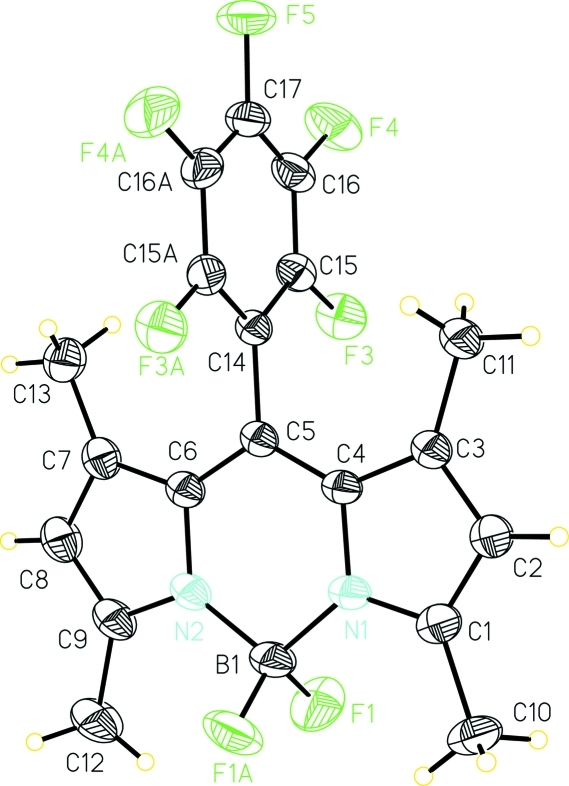
A view of the title compound with the atomic numbering scheme. Displacement ellipsoids for non-H atoms are drawn at the 30% probability level. 'A' labeled atoms are generated by symmetry code x, 1-y, z.

## supplementary crystallographic information

### Comment

Boron-dipyrromethene (BODIPY) dyes are excellent and famous fluorophores, with a high molar extinction coefficient and high fluorescence quantum yield, which have recently received considerable attention with regard to the design of fluorescence labels and biomolecular sensors (Bergström *et al.*, 2002; Trieflinger *et al.*, 2005). Here, the synthesis and the crystal structure of the title compound are reported. The observed geometric parameters are generally comparable with the reported values for other BODIPY-based compounds (Picou *et al.*, 1990; Wang *et al.*, 2007).

As shown in Fig. 1, the BODIPY skeleton of the molecule, which is formed from three fused heterocyclic rings, is planar, as this system lies on a mirror plane. The *sp*^3^-hybridized B centre appears as a slightly distorted tetrahedron, with N—B—N and F—B—F angles of 107.5 (2) and 109.8 (3)°. The two B—N distances are almost identical, implying the usual delocalization of the positive charge. The average bond lengths for B—N and B—F and the average N—B—N, F—B—F and F—B—N bond angles indicate a tetrahedral BF_2_N_2_ configuration and are in good agreement with previous published data (Kuhn, *et al.*, 1990; Picou *et al.*, 1990; Wang *et al.*, 2007). No unusual values are observed in the molecular structure. Perhaps due to the steric repulsion from the C11 and C13 methyl groups, the pentafluorophenyl ring is perpendicular to the BODIPY ring plane, with a dihedral angle constrained by symmetry to be 90°.

### Experimental

Pentafluorobenzaldehyde (2 mmol) and 2,4-dimethyl-1*H*-pyrrole (4 mmol) were dissolved in 50 ml of dry CH_2_Cl_2_ under an Ar atmosphere. One drop of trifluoroacetic acid (TFA) was added, and the solution was stirred at room temperature overnight. Thin layer chromatography (TLC) monitoring (silica; CH_2_Cl_2_) showed complete consumption of the aldehyde. At this point, a solution of dichlorodicyanobenzoquinone (DDQ, 2 mmol) in dry CH_2_Cl_2_ (20 ml) was added, and the mixture was stirred for additional 15 min. The reaction mixture was then treated with *N*,*N*-diisopropylethylamine (DIEA, 3 ml) and boron trifluoride etherate (3 ml). After stirring for another 30 min, the dark brown solution was washed with water (3×50 ml) and brine (50 ml), dried over Na_2_SO_4_, and concentrated under reduced pressure. The crude product was purified by silica-gel flash column chromatography and recrystallization from CHCl_3_/hexane. Single crystals suitable for X-ray analysis were obtained from an acetonitrile solution by slow evaporation.

### Refinement

Positional parameters of all the H atoms were calculated geometrically and were allowed to ride on the C atoms to which they are bonded. Isotropic displacement parameters for H atoms were refined.

### Crystal data

**Table:**

C_19_H_14_BF_7_N_2_	*F*(000) = 840
*M**_r_* = 414.13	*D*_x_ = 1.501 Mg m^−^^3^
Monoclinic, *C*2/*m*	Mo *K*α radiation, λ = 0.71073 Å
Hall symbol: -C 2y	Cell parameters from 2191 reflections
*a* = 12.4060 (5) Å	θ = 3.1–27.5°
*b* = 7.5490 (9) Å	µ = 0.14 mm^−^^1^
*c* = 19.720 (3) Å	*T* = 293 K
β = 97.12 (2)°	Prism, red
*V* = 1832.6 (4) Å^3^	0.2 × 0.2 × 0.2 mm
*Z* = 4	

### Data collection

**Table:**

Rigaku SCXmini diffractometer	1936 independent reflections
Radiation source: fine-focus sealed tube	1585 reflections with *I* > 2σ(*I*)
graphite	*R*_int_ = 0.032
Detector resolution: 13.6612 pixels mm^-1^	θ_max_ = 26.0°, θ_min_ = 3.1°
ω scans	*h* = −15→15
Absorption correction: multi-scan (*CrystalClear*; Rigaku, 2005)	*k* = −9→9
*T*_min_ = 0.973, *T*_max_ = 0.979	*l* = −24→24
8232 measured reflections	

### Refinement

**Table:**

Refinement on *F*^2^	Primary atom site location: structure-invariant direct methods
Least-squares matrix: full	Secondary atom site location: difference Fourier map
*R*[*F*^2^ > 2σ(*F*^2^)] = 0.056	Hydrogen site location: inferred from neighbouring sites
*wR*(*F*^2^) = 0.162	H-atom parameters constrained
*S* = 1.06	*w* = 1/[σ^2^(*F*_o_^2^) + (0.091*P*)^2^ + 0.8854*P*] where *P* = (*F*_o_^2^ + 2*F*_c_^2^)/3
1936 reflections	(Δ/σ)_max_ < 0.001
170 parameters	Δρ_max_ = 0.24 e Å^−^^3^
0 restraints	Δρ_min_ = −0.23 e Å^−^^3^
0 constraints	

### Fractional atomic coordinates and isotropic or equivalent isotropic displacement parameters (Å^2^)

**Table:**

	*x*	*y*	*z*	*U*_iso_*/*U*_eq_	
F1	0.12945 (15)	0.3502 (3)	0.07469 (8)	0.1040 (7)	
F3	0.28302 (13)	0.18847 (16)	0.34101 (7)	0.0721 (5)	
F4	0.35839 (14)	0.1888 (2)	0.47541 (8)	0.0928 (6)	
F5	0.39356 (18)	0.5000	0.54333 (9)	0.0988 (9)	
N1	0.08602 (19)	0.5000	0.17499 (11)	0.0486 (6)	
N2	0.2747 (2)	0.5000	0.14360 (11)	0.0523 (6)	
C1	−0.0234 (2)	0.5000	0.17144 (15)	0.0564 (7)	
C2	−0.0523 (2)	0.5000	0.23784 (16)	0.0597 (8)	
H2A	−0.1229	0.5000	0.2490	0.104 (14)*	
C3	0.0406 (2)	0.5000	0.28399 (14)	0.0489 (7)	
C4	0.1291 (2)	0.5000	0.24405 (12)	0.0446 (6)	
C5	0.2418 (2)	0.5000	0.26194 (12)	0.0422 (6)	
C6	0.3151 (2)	0.5000	0.21363 (13)	0.0475 (6)	
C7	0.4316 (2)	0.5000	0.21939 (16)	0.0563 (7)	
C8	0.4565 (3)	0.5000	0.15330 (17)	0.0682 (9)	
H8A	0.5263	0.5000	0.1408	0.080 (11)*	
C9	0.3606 (3)	0.5000	0.10769 (15)	0.0627 (8)	
C10	−0.0981 (3)	0.5000	0.10532 (18)	0.0783 (11)	
H10A	−0.0559	0.5000	0.0677	0.16 (2)*	
H10B	−0.1430	0.6038	0.1031	0.23 (3)*	
C11	0.0428 (3)	0.5000	0.36050 (15)	0.0629 (9)	
H11A	−0.0302	0.5000	0.3719	0.079 (11)*	
H11B	0.0801	0.6038	0.3792	0.093 (9)*	
C12	0.3492 (4)	0.5000	0.03121 (18)	0.0875 (13)	
H12A	0.2735	0.5000	0.0135	0.25 (4)*	
H12B	0.3833	0.3962	0.0156	0.172 (19)*	
C13	0.5127 (3)	0.5000	0.28225 (19)	0.0693 (9)	
H13A	0.5847	0.5000	0.2693	0.126 (17)*	
H13B	0.5027	0.3962	0.3089	0.129 (13)*	
C14	0.2846 (2)	0.5000	0.33641 (13)	0.0425 (6)	
C15	0.30342 (16)	0.3443 (3)	0.37278 (10)	0.0490 (5)	
C16	0.34105 (17)	0.3429 (3)	0.44160 (11)	0.0602 (6)	
C17	0.3592 (2)	0.5000	0.47584 (15)	0.0638 (9)	
B1	0.1532 (3)	0.5000	0.11399 (17)	0.0612 (9)	

### Atomic displacement parameters (Å^2^)

**Table:**

	*U*^11^	*U*^22^	*U*^33^	*U*^12^	*U*^13^	*U*^23^
F1	0.0923 (11)	0.1505 (17)	0.0710 (10)	−0.0214 (11)	0.0177 (8)	−0.0597 (10)
F3	0.0976 (11)	0.0457 (7)	0.0730 (9)	0.0076 (7)	0.0110 (7)	0.0018 (6)
F4	0.1028 (12)	0.1033 (12)	0.0732 (10)	0.0309 (10)	0.0147 (8)	0.0448 (9)
F5	0.0785 (14)	0.177 (3)	0.0379 (10)	0.000	−0.0059 (9)	0.000
N1	0.0560 (13)	0.0546 (13)	0.0346 (11)	0.000	0.0027 (9)	0.000
N2	0.0601 (14)	0.0615 (14)	0.0377 (12)	0.000	0.0155 (10)	0.000
C1	0.0547 (17)	0.0633 (18)	0.0491 (16)	0.000	−0.0017 (12)	0.000
C2	0.0491 (16)	0.079 (2)	0.0516 (16)	0.000	0.0085 (13)	0.000
C3	0.0495 (15)	0.0576 (16)	0.0405 (14)	0.000	0.0086 (11)	0.000
C4	0.0520 (15)	0.0477 (14)	0.0337 (12)	0.000	0.0041 (10)	0.000
C5	0.0507 (14)	0.0386 (13)	0.0380 (13)	0.000	0.0081 (11)	0.000
C6	0.0535 (15)	0.0490 (15)	0.0408 (14)	0.000	0.0089 (11)	0.000
C7	0.0541 (16)	0.0601 (17)	0.0570 (17)	0.000	0.0157 (13)	0.000
C8	0.0590 (19)	0.087 (2)	0.064 (2)	0.000	0.0271 (16)	0.000
C9	0.075 (2)	0.071 (2)	0.0468 (16)	0.000	0.0246 (15)	0.000
C10	0.069 (2)	0.108 (3)	0.0514 (19)	0.000	−0.0157 (16)	0.000
C11	0.0517 (16)	0.095 (3)	0.0437 (15)	0.000	0.0130 (13)	0.000
C12	0.099 (3)	0.121 (4)	0.0486 (19)	0.000	0.033 (2)	0.000
C13	0.0507 (17)	0.088 (3)	0.069 (2)	0.000	0.0068 (15)	0.000
C14	0.0428 (13)	0.0479 (14)	0.0375 (13)	0.000	0.0077 (10)	0.000
C15	0.0507 (10)	0.0498 (11)	0.0474 (10)	0.0060 (9)	0.0099 (8)	0.0030 (8)
C16	0.0533 (11)	0.0795 (16)	0.0488 (11)	0.0128 (11)	0.0104 (9)	0.0208 (11)
C17	0.0490 (16)	0.104 (3)	0.0380 (14)	0.000	0.0051 (12)	0.000
B1	0.068 (2)	0.082 (2)	0.0346 (15)	0.000	0.0083 (14)	0.000

### Geometric parameters (Å, °)

**Table:**

B1—F1	1.382 (3)	C7—C8	1.376 (4)
B1—N1	1.546 (4)	C7—C13	1.497 (5)
B1—N2	1.548 (5)	C8—C9	1.400 (5)
F3—C15	1.342 (3)	C8—H8A	0.9301
F4—C16	1.345 (3)	C9—C12	1.497 (4)
F5—C17	1.346 (3)	C10—H10A	0.9598
N1—C1	1.350 (4)	C10—H10B	0.9600
N1—C4	1.400 (3)	C11—H11A	0.9601
N2—C9	1.350 (4)	C11—H11B	0.9600
N2—C6	1.409 (4)	C12—H12A	0.9600
C1—C2	1.400 (4)	C12—H12B	0.9601
C1—C10	1.503 (4)	C13—H13A	0.9600
C2—C3	1.377 (4)	C13—H13B	0.9600
C2—H2A	0.9298	C14—C15	1.382 (2)
C3—C4	1.428 (4)	C14—C15^i^	1.382 (2)
C3—C11	1.506 (4)	C15—C16	1.379 (3)
C4—C5	1.398 (4)	C16—C17	1.370 (3)
C5—C6	1.397 (4)	C17—C16^i^	1.370 (3)
C5—C14	1.498 (3)	B1—F1^i^	1.382 (3)
C6—C7	1.435 (4)		
			
C1—N1—C4	108.1 (2)	C8—C9—C12	127.9 (3)
C1—N1—B1	126.5 (2)	C1—C10—H10A	109.6
C4—N1—B1	125.4 (2)	C1—C10—H10B	109.4
C9—N2—C6	107.8 (3)	H10A—C10—H10B	109.5
C9—N2—B1	126.6 (3)	C3—C11—H11A	109.5
C6—N2—B1	125.5 (2)	C3—C11—H11B	109.5
N1—C1—C2	108.9 (3)	H11A—C11—H11B	109.5
N1—C1—C10	123.6 (3)	C9—C12—H12A	109.5
C2—C1—C10	127.6 (3)	C9—C12—H12B	109.5
C3—C2—C1	109.2 (3)	H12A—C12—H12B	109.5
C3—C2—H2A	125.5	C7—C13—H13A	109.4
C1—C2—H2A	125.4	C7—C13—H13B	109.5
C2—C3—C4	105.8 (2)	H13A—C13—H13B	109.5
C2—C3—C11	124.9 (3)	C15—C14—C15^i^	116.6 (2)
C4—C3—C11	129.2 (3)	C15—C14—C5	121.69 (13)
N1—C4—C5	119.6 (2)	C15^i^—C14—C5	121.69 (13)
N1—C4—C3	108.0 (2)	F3—C15—C14	119.56 (18)
C5—C4—C3	132.3 (2)	F3—C15—C16	118.27 (19)
C6—C5—C4	122.9 (2)	C14—C15—C16	122.2 (2)
C6—C5—C14	119.1 (2)	F4—C16—C17	119.9 (2)
C4—C5—C14	118.0 (2)	F4—C16—C15	120.6 (2)
C5—C6—N2	119.1 (2)	C17—C16—C15	119.5 (2)
C5—C6—C7	132.9 (3)	F5—C17—C16	119.99 (14)
N2—C6—C7	108.0 (2)	F5—C17—C16^i^	119.99 (14)
C8—C7—C6	105.5 (3)	C16—C17—C16^i^	120.0 (3)
C8—C7—C13	125.3 (3)	F1—B1—F1^i^	109.8 (3)
C6—C7—C13	129.2 (3)	N1—B1—N2	107.5 (2)
C7—C8—C9	109.6 (3)	F1—B1—N1	109.8 (2)
C7—C8—H8A	125.2	F1^i^—B1—N1	109.8 (2)
C9—C8—H8A	125.1	F1—B1—N2	110.0 (2)
N2—C9—C8	109.0 (3)	F1^i^—B1—N2	110.0 (2)
N2—C9—C12	123.1 (3)		

Symmetry codes: (i) *x*, −*y*+1, *z*.
